# Oxygen Reduction Reaction Activity of Microwave Mediated Solvothermal Synthesized CeO_2_/g-C_3_N_4_ Nanocomposite

**DOI:** 10.3389/fchem.2019.00403

**Published:** 2019-06-06

**Authors:** Siba Soren, Ipsha Hota, A. K. Debnath, D. K. Aswal, K. S. K. Varadwaj, Purnendu Parhi

**Affiliations:** ^1^Department of Chemistry, Ravenshaw University, Cuttack, India; ^2^Technical Physics Division, Bhabha Atomic Research Center, Mumbai, India

**Keywords:** g-C_3_N_4_, CeO_2_, cyclic voltammogram, linear sweep voltammetry, rotating disk electrode, rotating ring disk electrode

## Abstract

Electrocatalytic active species like transition metal oxides have been widely combined with carbon-based nanomaterials for enhanced Oxygen Reduction Reaction (ORR) studies because of the synergistic effect arising between different components. The aim of the present study is to synthesize CeO_2_/g-C_3_N_4_ system and compare the ORR activity with bare CeO_2_. Ceria (CeO_2_) embedded on g-C_3_N_4_ nanocomposite was synthesized by a single-step microwave-mediated solvothermal method. This cerium oxide-based nanocomposite displays enhanced ORR activity and electrochemical stability as compared to bare ceria.

## Introduction

Metal–air batteries and fuel cells are alternative energy transfer devices designed to meet the requirements of sustainable energy (Sun et al., 2017). Direct Methanol Fuel Cells (DMFCs) are documented as an ideal candidate for laptop, mobile, and digital camera applications. Efficient reduction of O_2_ to water is a major challenge in energy conversion in DMFCs. The Oxygen Reduction Reaction (ORR) in alkaline DMFCs proceeds via a 4-electron pathway, (O_2_ + 2H_2_O + 4e^−^ → 4OH^−^) which is preferable over the 2-electron pathway (O_2_ + H_2_O + 2e^−^ → ${\text{HO}}_{2}^{-}+$ OH^−^) (Zhang and Song, 2008). The 2-electron process is unfavorabl because of the production of corrosive peroxide species, which can cause degradation of electrochemical cells. The precious metal catalysts such as Pt and Pt- based alloys used to catalyze the ORR process are expensive and available in limited quantity (Peng and Yang, 2009; Kim et al., 2010). These Pt-based catalysts are also intolerant to methanol, which is used as fuel in DMFCs. Design of new non-precious electrocatalyst with improved ORR activity is still a challenge before the scientific community. First row transition metal oxides (TMOs) (Bashyam and Zelenay, 2006; Cheng et al., 2009; Jaouen et al., 2011; Cheng and Chen, 2012) have already been used as robust alternatives for promoting the ORR in alkaline conditions. The low electrical conductivity of the TMOs influences the electron transfer process in ORR (Soren et al., 2016).

Carbon materials possess a specific place for the ORR in DMFCs. Various carbon materials like graphite (Jiao et al., 2014), carbon black, carbon nanotube, and activated carbon are mostly used as supporting materials in the preparation of electrocatalysts due to their high electrical conductivity, corrosion resistance, porous structure and specific surface area (Liang et al., 2011). TMOs when embedded with reduced graphene oxide show enhanced catalytic performance because of a synergetic effect between TMOs and graphene oxide (Liang et al., 2011, 2012; Wang et al., 2011; Guo and Sun, 2012; Guo et al., 2012; Wu et al., 2015).

Hetero atoms (e.g., N, B, P, S, and I) were also doped in the reduced graphene oxide in order to improve the catalytic active sites in reduced graphene oxide (Behnam, 2017). In the recent past, nitrogen-doped graphene oxide has become a potential carbon-based electrocatalyst for ORR because of its low cost, high stability, and high efficiency (Qu et al., 2010; Geng et al., 2011; Yang et al., 2012; Paraknowitsch and Thomas, 2013). The electronic environment of doping nitrogen on a reduced graphene sheet in three configurations (e.g., pyridynic, pyrrolic, and graphitic nitrogen) induces an uneven charge distribution in adjacent sites, and as a result it alters the local spin or charge density. It promotes oxygen adsorption and helps in the enhancement of ORR performance (Ikeda et al., 2008; Niwa et al., 2009; Liu et al., 2010; Qu et al., 2010; Rao et al., 2010; Kim et al., 2011; Li et al., 2011, 2013; Sheng et al., 2011; Zhang and Xia, 2011; Lai et al., 2012; Parvez et al., 2012; Sharifi et al., 2012; Wang et al., 2012; Zhang et al., 2013; Zheng et al., 2013; Bag et al., 2014). Transition metal oxide embedded in N doped carbon systems is reported as a promoter of ORR catalytic activity by facilitating electron transfer (Bag et al., 2014). Rare earth oxide-based systems are now extensively studied for ORR due to their unique electronic structure, bonding characteristics and variable oxidation states. There are very few reports available in the literature where rare earth oxides such as lanthanum oxide, samarium oxide, and cerium oxide have been studied for their ORR properties (Soren et al., 2016; Wang et al., 2016, 2017). The unique structural properties of CeO_2_ have contributed toward the promising electrocatalytic activity of CeO_2_. However, poor electronic conductivity of CeO_2_ limits its application toward ORR. Thus, in order to enhance the electrocatalytic activity, CeO_2_ is doped with different metals or embedded with a conductive active framework. Recently, our group has published an article on ORR activity of the CeO_2_/NrGO system (Soren et al., 2016).

Researchers have found a new material analogy to N-doped rGO called graphitic carbon nitride (g-C_3_N_4_) (Qiao et al., 2016). It is a carbon- and nitrogen-based polymeric material. Graphitic carbon nitride (g-C_3_N_4_) is a nitrogen-rich carbon-based material. But as reported in the literature g-C_3_N_4_ is an inert electrocatalyst (Liu and Zhang, 2013; Zou et al., 2013). However, g-C_3_N_4_ with metal doping or metal oxide doping has already been reported as a promising electrocatalyst for ORR and OER (Oxygen Evolution Reaction), when embedded with transition metal/metal oxide (Liu and Zhang, 2013; Zou et al., 2013).

It is expected that CeO_2_/g-C_3_N_4_ composite can enhance the ORR in fuel cells. In this paper, we have investigated the ORR activity of CeO_2_ embedded with g-C_3_N_4_ to show whether it follows a 2-electron or 4-electron pathway in the ORR process. The hybrid CeO_2_/g-C_3_N_4_ nanostructures were prepared by the microwave mediated polyol method.

## Materials and Methods

### Chemicals

Melamine, Ammonium Cerium (IV) Nitrate and 1, 4-Butanediol were procured from HIMEDIA. All the chemicals were used as received.

### Synthesis of g-C_3_N_4_

About 3 g of melamine was taken in china crucible and was heated for 4 h at 520°C with a moderate heating rate of 10°C/min inside the muffle furnace. The product obtained was cooled to room temperature. The prepared g-C_3_N_4_ was characterized for further work.

### Synthesis of CeO_2_/g-C_3_N_4_ Composite

In typical synthesis 50 mg of the above-prepared g-C_3_N_4_ was added to 20 ml of 1, 4-butanediol. The solution was stirred at 300 rpm in a 50 ml beaker to make a heterogeneous mixture. A total of 250 mg of ammonium cerium (IV) nitrate was added to this heterogeneous solution and again stirred at the same rpm for 10 min until the color of the solution changed from light yellow to orange red. The entire solution was transferred to a Teflon vessel. The vessel was tightly sealed and irradiated with microwave radiation (MDS 6) for 10 min at 180°C. The reaction mixture was allowed to cool to room temperature after completion of the reaction. The obtained light yellow-colored precipitate was centrifuged several times with distilled water, ethanol, and acetone to remove the impurities. Finally, the product was kept for drying in an oven over night at 60°C.

### Characterization

The crystallographic phases were identified by XRD measurements using a Rigaku Ultima-IV Advance X-ray Diffractometer operating at 40 KV (radiation source Cu Kα, wavelength = 1.5418 Å). FTIR analysis was carried out with the help of Thermo Fischer Nicolet iS5 FTIR instrument. The XPS measurement was performed using DESA-150 electron analyzer (Staib (1253.6 eV) as radiation source. Transmission Electron Microscope (TEM) and High Resolution Transmission Electron Microscope (HRTEM) images were obtained by the Model FEI Technai G2 S-Twin (Benson et al., 2014). Electrochemical Impedance Spectroscopic analysis was recorded CH660E electrochemical work station.

### Electrochemical Measurements

The electrochemical measurements were conducted in a conventional three-electrode system using a Metrohm Autolab 204 B.V. (Metrohm Autolab, Netherland). During the measurements, saturated Ag/AgCl electrode, Pt wire, and modified glassy carbon (GC) electrode were used as reference, counter, and working electrode respectively. Synthesized electrocatalyst material was loaded on a pre-cleaned GC electrode during preparation of the working electrode. For the preparation of catalytic ink 5 mg of as synthesized electrocatalyst was dispersed in 2 ml isopropanol, 3 ml double distilled H_2_O, and 25 micro liter Nafion solution (as binder) in an ultrasonic bath for 30 min (Soren et al., 2016). About 12 μl of well-dispersed catalytic ink (~0.012 mg) was drop casted onto the polished GC electrode of surface area (0.07067 cm^2^). The modified glassy carbon electrode was dried in a vacuum oven for 3 h. About 33.3 μl of previously prepared catalyst ink was drop casted on RRDE electrode (GC disk electrode) with 5 mm diameter (S = 0.196 cm^2^) to make the loading the same as in RDE studies.

## Result and Discussion

### Composition and Structure Characterization of CeO_2_/gC_3_N_4_ Hybrid

**Figure 1:**
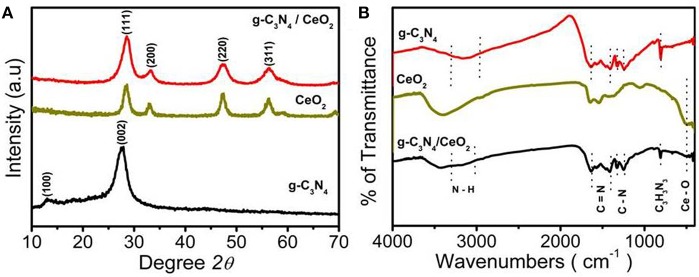
**(A)** XRD of g-C_3_N_4_, CeO_2_, CeO_2_/ g-C_3_N_4_ and **(B)** FTIR spectra of g-C_3_N_4_, CeO_2_, CeO_2_/g-C_3_N_4_.

XRD patterns of the synthesized composite (1:1) were studied and compared with the XRD pattern of individual CeO_2_ and g-C_3_N_4_ (Ferrero et al., 2016). In the case of a pure g-C_3_N_4_ sample, a strong diffraction peak appears at 27.61° that corresponds to the (002) plane. The strong diffraction peak arises because of the stacking of the conjugated aromatic system (Wang et al., 2009). The sharp peak at 27.61° indicates tight packing in g-C_3_N_4_, which is because of the strong binding between the layers and large localization of electrons. Another small peak at 13.12° can be assigned to the (100) plane. The diffraction peaks of the pure cubic fluorite structure of CeO_2_ were indexed to the (111), (200), (220), and (311) planes (JCPDS 81-0792) (Soren et al., 2015). After the incorporation of CeO_2_ into the g-C_3_N_4_ network, the XRD pattern shows an absence of peak at 13.12° even though the weight ratio of g-C_3_N_4_ and CeO_2_ are nearly 1:1 in the composite (Liu and Zhang, 2013). The peak corresponding to the (111) plane of CeO_2_, which coincides with the highest intensity peak of bare g-C_3_N_4_, has been shifted to higher 2θ value, and the intensity of the peak has also been increased. From these findings it can be concluded that CeO_2_ nanoparticles are successfully embedded into in-planes of g-C_3_N_4_ sheets (Thomas et al., 2008; Zhang et al., 2008; Xu et al., 2013) (Figure 1A).

The chemical structures of the samples were evaluated by the FTIR analysis. A broad band between 3,000 and 3,500 cm^−1^ was noticed in the composite, which can be attributed to stretching vibration of N-H and surface adsorbed water molecules. The absorption peaks at 1,572 and 1,632 cm^−1^ (Li et al., 2015) were due to C = N stretching (Bojdys et al., 2008; Yan et al., 2009) while for aromatic C-N stretching, peaks at 1,253, 1,320, and 1,425 cm^−1^ were observed. The main structural peaks at 808 cm^−1^ corresponded to the breathing mode of triazine units of g-C_3_N_4_ (Xu et al., 2013), which reveals that the graphitic C-N network of g-C_3_N_4_ was not affect even after the inclusion of CeO_2_ in the g-C_3_N_4_ layer (Figure 1B).

**Figure 2:**
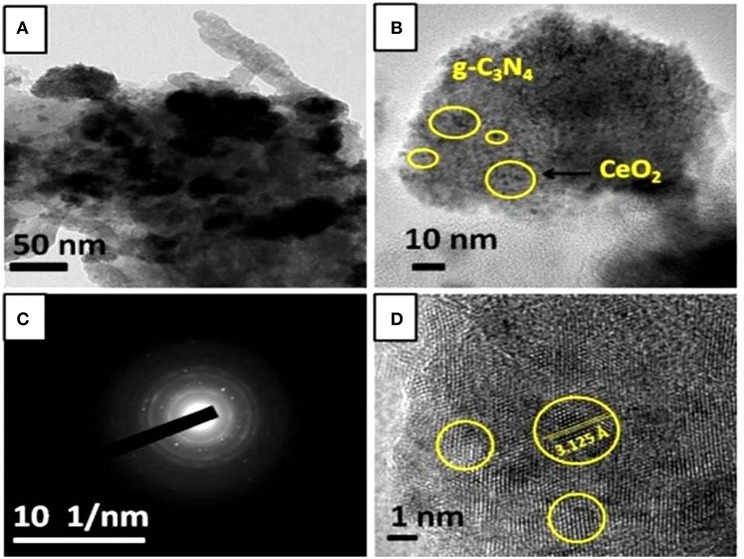
**(a)** TEM, **(b)** HRTEM at 10 nm, **(c)** SAED, and **(d)** HRTEM at 1 nm of CeO_2_/g-C_3_N_4_.

CeO_2_/g-C_3_N_4_ composite showed a layered structure of g-C_3_N_4_ (Figure 2a), and several small dark images of CeO_2_ nanoparticle appeared in the TEM image (Figure 2b). The appearance of some bright spots and the diffuse rings in the SAED pattern of CeO_2_/g-C_3_N_4_ corresponds to the growth of crystalline CeO_2_ nanoparticle on the amorphous g-C_3_N_4_ sheet (Figure 2c). The fringe spacing was measureed to be 3.125 Å, which corresponds to the (111) lattice plane of the cubic fluorite CeO_2_ structure (Figure 2d).

**Figure 3:**
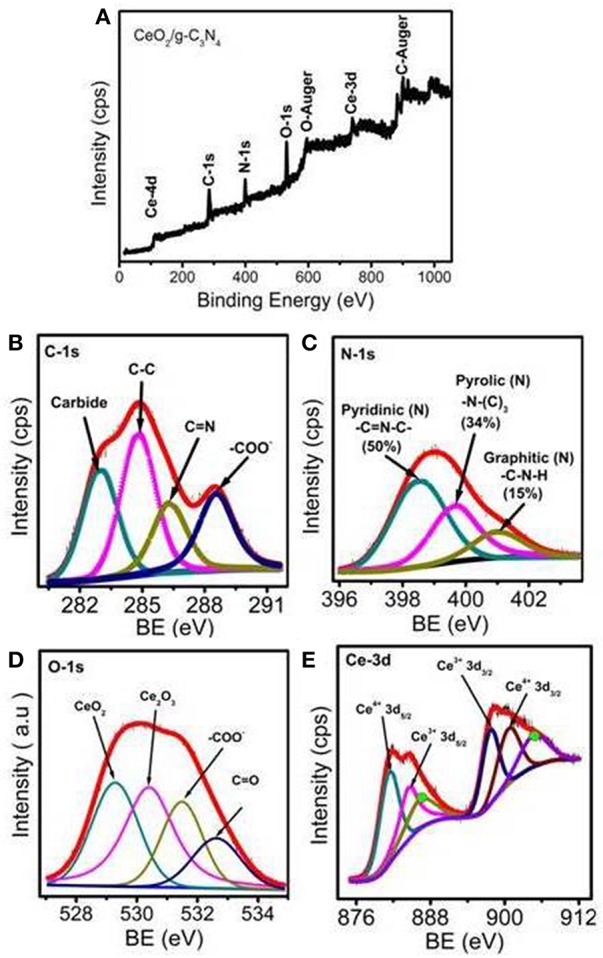
**(A)** XPS survey scan of CeO_2_/ g-C_3_N_4_, Deconvoluted XPS spectrum of **(B)** C 1s, **(C)** N 1s, **(D)** O 1s, **(E)** Ce 3d.

The composition and chemical structure of the synthesized material were established by X-ray photoelectron spectroscopy. The high resolution XPS survey spectra of C 1s, N 1s, O 1s, and Ce 3d XPS spectra of CeO_2_/g-C_3_N_4_ (1:1) composite is shown in Figure 3A. The XPS spectra of the C 1s core level for CeO_2_/g-C_3_N_4_ can be deconvoluted into four components including the standard reference carbon (283 eV) (Xing et al., 2014). The peak at 284.8 eV corresponds to sp^2^-bonded C-C (Raymundo-Pinero et al., 2002). The peaks at 286.3 and 288.5 eV (Guan et al., 2018) are ascribed to C = N and N-C-N in g-C_3_N_4_ respectively (Figure 3B) (Yan et al., 2012). The main peak of N 1s at 398.5 eV is assigned to sp^2^ nitrogen (C=N-C) (pyridinic) present in triazine rings, while the peak at 399.6 eV arises from the tertiary nitrogen bonded to carbon atoms in the form of N–(C)_3_ (pyrollic). The peak at 401.1 eV can be ascribed to g (C-N-H) (ghaphitic) (Figure 3C) (Raymundo-Pinero et al., 2002). The % of N in total spectrum is calculated to be 7.09%. The percentages of different levels of nitrogen in the system were 50.6, 34.48, and 15.05% for pyridinic, pyrrolic, and graphitic nitrogen, respectively. The O 1s spectrum is deconvoluted at 529.2, 531.0, and 533.2 eV for CeO_2_, COOH and OH respectively. These spectrums suggest the formation of CeO_2_ on g-C_3_N_4_ (Figure 3D). The oxidation states of Ce in g-C_3_N_4_/CeO_2_ composites were examined by deconvolution of Ce 3d peaks. The two peaks at 881.6 and 884.7 eV can be attributed to 3d_5/2_ of Ce^4+^ and Ce^3+^ core electrons respectively. Further peaks at 897.5 and 900.1 eV can be ascribed to 3d_3/2_ of Ce^3+^ and Ce^4+^, respectively (Figure 3E) (Zheng et al., 2017).

### Electrochemical Performance of CeO_2_/gC_3_N_4_ Hybrid

**Figure 4:**
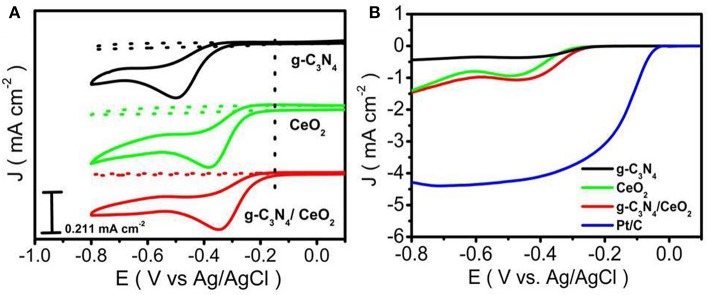
**(A)** CV study of g-C_3_N_4_, CeO_2_, CeO_2_/g-C_3_N_4_, **(B)** LSV study of g-C_3_N_4_, CeO_2_, CeO_2_/g-C_3_N_4_ and Pt/C at 1,600 rpm.

**Figure 5:**
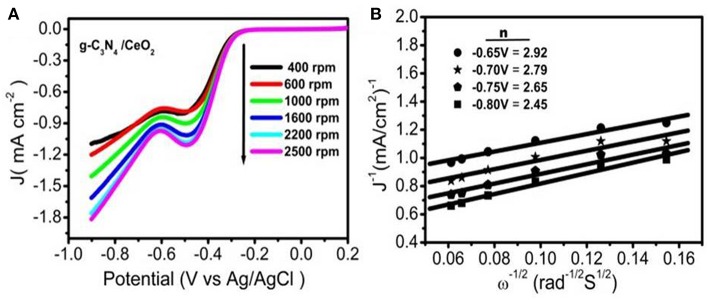
**(A)** RDE of CeO_2_/g-C_3_N_4_ at various rotation rates, **(B)** K-L plot of CeO_2_/g-C_3_N_4_ at different potentials.

The cyclic voltammogram (CV) was performed in N_2_ as well as O_2_ saturated 0.1 M KOH solution for the three materials (g-C_3_N_4_, CeO_2_ and CeO_2_/ g-C_3_N_4_) in the potential range of −0.8 to 0.2 V at various scan rates (Ferrero et al., 2016). No reduction peaks were observed in the N_2_-saturated condition. After O_2_ was introduced for 30 min, intense reduction peaks of g-C_3_N_4_ (Jiao et al., 2014), CeO_2_, and CeO_2_/g-C_3_N_4_ at E_onset_ −0.3, −0.24, and −0.17 V vs. Ag/AgCl, respectively, were recorded (Figure 4A). To investigate ORR performance, linear sweep voltammetry (LSV) was recorded for the prepared materials together with commercial 20 wt% Pt/C, in O_2_ saturated 0.1 M KOH solution using Rotating Disk Electrode (RDE) at 1,600 RPM (Wu X. et al., 2017). From the LSV, plot the onset potential was observed to be −0.3, −0.23, and −0.2 V vs. Ag/AgCl for g-C_3_N_4_, CeO_2_, and CeO_2_/g-C_3_N_4_, respectively (Figure 4B). The shift in onset potential of 30 mV for CeO_2_/g-C_3_N_4_ as compared to CeO_2_ indicates a synergetic interaction between CeO_2_ and g-C_3_N_4_ in the composite which facilitates ORR, but the constancy in the current value of CeO_2_/g-C_3_N_4_ composite with CeO_2_ indicates a slow ORR rate. A positive shift of onset potential (100 mV) between CeO_2_/g-C_3_N_4_ (E_onset_ = −0.2V vs. Ag/AgCl) and g-C_3_N_4_ (E_onset_ = −0.3V vs. Ag/AgCl) suggests a strong interaction between CeO_2_ and g-C_3_N_4_. We observed a small improvement in the E_1/2_ for CeO_2_/g-C_3_N_4_ composite (E_1/2_ is −0.383 V) as compared to their individual counterparts (E_1/2_ is −0.383 V and −0.388 V for bare CeO_2_ and g-C_3_N_4_, respectively) (Ferrero et al., 2016). LSVs of CeO_2_/g-C_3_N_4_ at different rotation speeds from 400 to 2,500 rpm were carried out with Rotating Disk Electrode (RDE) to study electron transfer kinetics during ORR process (Figure 5A). It is observed that with an increase in rotation rate the diffusion rate of oxygen molecules also increases, which leads to a gradual increase in current density value. Koutecky–Levich (K–L) plots were plotted (Figure 5B) in order to gain better insight into the electron transfer process during ORR. The Koutecky–Levich (K–L) equation is given as follows:

(1) $$\begin{array}{l} 1/\text{J}={1/\text{J}}_{\text{L}}+{1/\text{J}}_{\text{K}}=\left(1/\text{B}\right)\omega^{-1/2}+{1/\text{J}}_{\text{K}} \end{array}$$

(2) $$\begin{array}{l} \text{B}={0.62\text{nFC}}_{0}{\left({\text{D}}_{0}\right)}^{2/3}\nu^{1/6} \end{array}$$

Here J is the measured current density, J_L_ and J_K_ are the diffusion and kinetic current densities, respectively, n is the transferred electron number per O_2_ molecule, ω is the angular velocity, F is the Faraday constant (F = 96,485 C mol^−1^), D_0_ is the O_2_ diffusion coefficient (1.9 × 10^−5^ cm^2^ s^−1^), C_0_ is the bulk concentration of O_2_ (1.2 × 10^−3^ mol L^−1^), ν is the kinematic viscosity of the electrolyte (0.01 m^2^ s^−1^) (Soren et al., 2016). At various electrode potentials J^−1^ vs. ω^−1/2^ graphs were plotted for CeO_2_/g-C_3_N_4_ (Figure 5B). The number of electrons transferred per O_2_ molecule (n) was calculated from the slopes of the best fit lines (Soren et al., 2016). The n value for the CeO_2_/g-C_3_N_4_ nano-composite was calculated around 3, which suggests the ORR kinetics proceeds through the 2-electron pathway.

**Figure 6:**
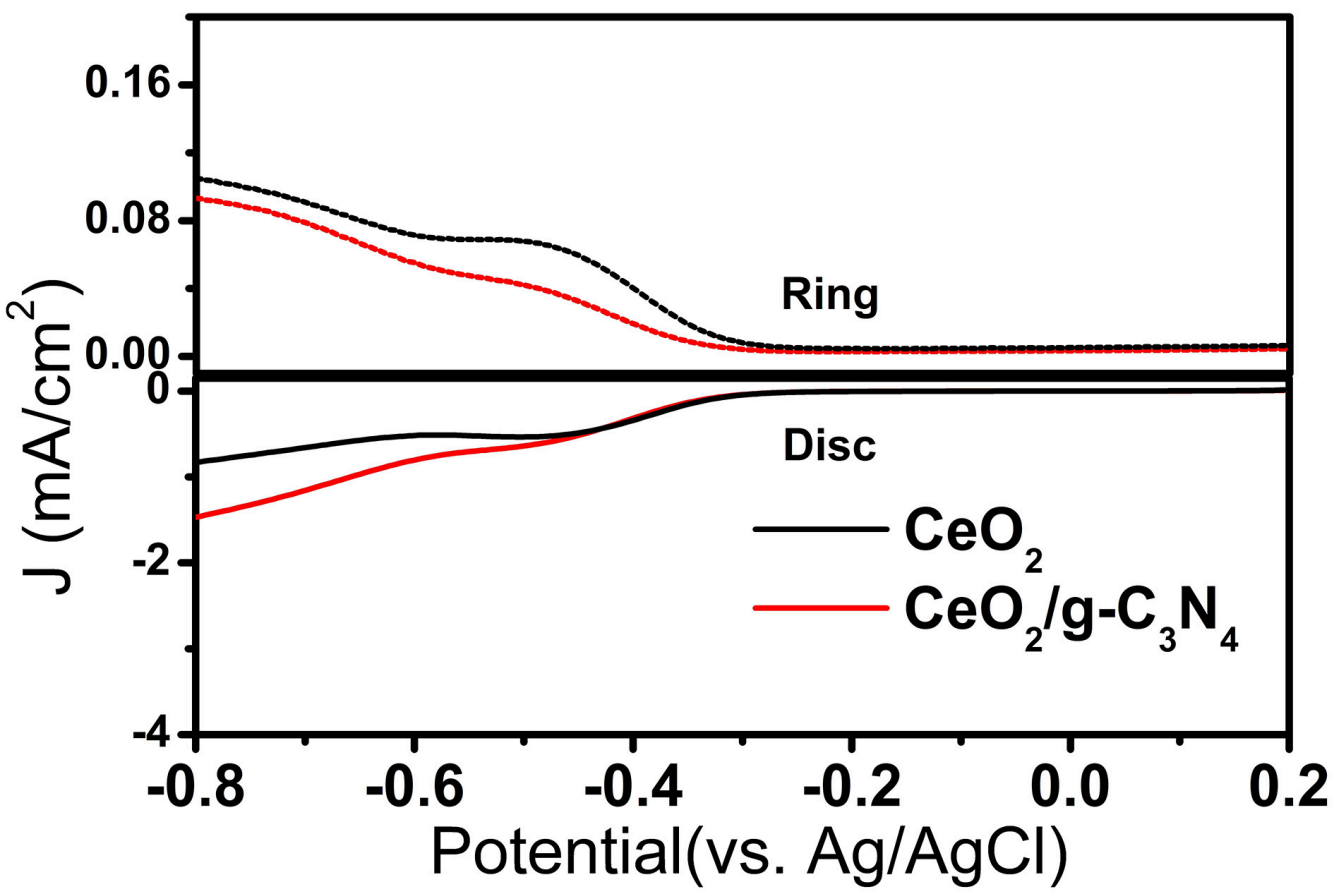
RRDE Comparison of CeO_2_ and CeO_2_/g-C_3_N_4_ systems.

To gain more information on the ORR mechanism, the electron transfer number ‘*n*’ and the rate of peroxide formation can be determined from the RRDE analysis (Ge et al., 2015). In RRDE, the ORR takes place at the GC disk (where the different catalysts were deposited) and the concentric Pt ring detects the peroxide production. Figure 6 shows the disk and ring currents for the CeO_2_ and CeO_2_/g-C_3_N_4_ systems. Both catalysts generate ring current at the onset potential for the ORR. All three electrodes display large disk currents with relatively low ring current. Here, n is the number of electrons transferred, I_D_ is the current measured at the GC disk, and I_R_ is the current measured at the Pt ring obtained through RRDE (Qiao et al., 2016). The value of “*N*” is 0.25, denoting the collection efficiency which is a design parameter provided by the RRDE manufacturer. The following two equations are used to determine electron transfer number and amount of peroxide produced during ORR (Ge et al., 2015).

(3) $$\begin{array}{l} \text{n}=\frac{4{\text{I}}_{\text{D}}}{{\text{I}}_{\text{D}}+\left({\text{I}}_{\text{R}}/\text{N}\right)} \end{array}$$

(4) $$\begin{array}{l} \;\%{\text{HO}}_{2}^{-}=\frac{200\left({\text{I}}_{\text{R}}/\text{N}\right)}{{\text{I}}_{\text{D}}+\left({\text{I}}_{\text{R}}/\text{N}\right)} \end{array}$$

**Figure 7:**
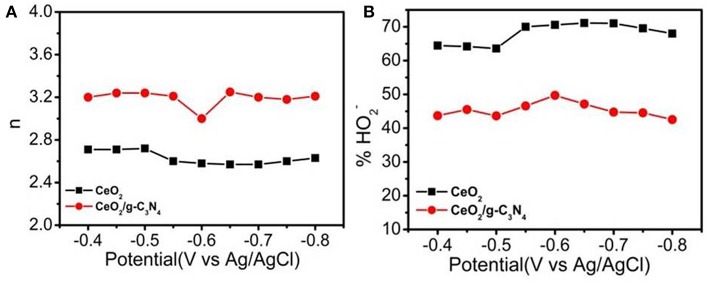
**(A)** Electron transfer number, **(B)** % of ${\text{HO}}_{2}^{-}$ formation of CeO_2_ and CeO_2_/g-C_3_N_4_.

At lower over potential regions (−0.4V vs. Ag/AgCl), the average value of “n” for CeO_2_, CeO_2_/g-C_3_N_4_ were 2.71, 3.2, respectively. The peroxide formation follows the reverse trend (CeO_2_ > CeO_2_/g-C_3_N_4_). At higher over potential regions (−0.6 V vs. Ag/AgCl) the “n” of CeO_2_ and CeO_2_/g-C_3_N_4_ remains constant, i.e., 2.57 and 3.2 respectively (Figure 7A). This indicates at the quasi-4-electron process followed CeO_2_/g-C_3_N_4_. The peroxide formation follows the reverse trend (CeO_2_ > CeO_2_/g-C_3_N_4_) at higher over potential regions (Figure 7B).

An enhancement of reaction kinetics toward the 4-electron ORR pathway is clearly observed from CeO_2_ to CeO_2_/ g-C_3_N_4_. The ORR activity can be ascribed to the oxygen vacancies in the CeO_2_ lattice, which originated from the mixed valence states. The defects can be easily healed by oxygen adsorption when exposed to oxygen environment (Soren et al., 2016). The improved ORR activity of CeO_2_/gC_3_N_4_ can be explained by considering the synergistic effect between CeO_2_ and carbon network containing different types of nitrogen (Pyridinc, pyrolic, and graphitic). Ruoff's group in their recent publication has confirmed the effect of nitrogen doping on the ORR activity (Lai et al., 2012). They have concluded that the catalytic activity is dependent on the nature and amount of nitrogen present in the matrix. It has been established that presence of graphitic nitrogen increases the limiting current whereas the pyridinic nitrogen alters the onset potential of ORR (Soren et al., 2016). Li et al. have shown that the direct reduction pathway for ORR is favored by the presence of pyridinic nitrogen (Li et al., 2013). Bag et al. also confirmed the vital role of pyridinic nitrogen in the enhancement of ORR activity (Bag et al., 2014). In this paper the XPS analysis reveals that CeO_2_/g-C_3_N_4_ has 50.6% pyridinic nitrogen. This explains the shift in onset potential while going from CeO_2_ to CeO_2_/gC_3_N_4_ composite (as discussed in Figure 4B)

**Figure 8:**
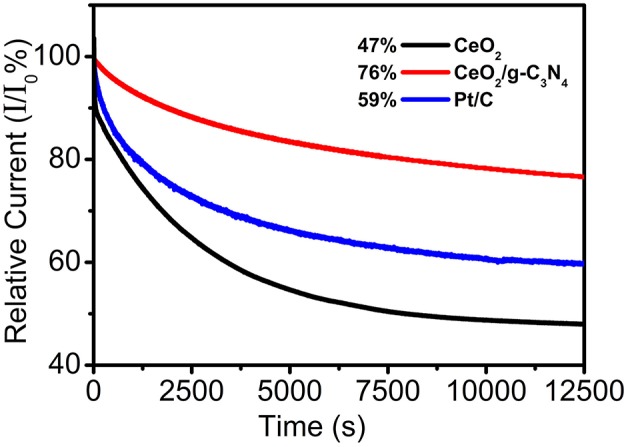
(Current–time) chronoamperometric responses for ORR on CeO_2_, CeO_2_/g-C_3_N_4_ and commercial Pt/C at −0.35 V at a rotational rate of 1,000 rpm.

Stability of electrocatalysts is another key parameter in the evaluation of their catalytic performance. The catalytic stability of CeO_2_/g-C_3_N_4_ along with commercial Pt/C and bare CeO_2_ were measured and compared by the Chronoamperometric response method at −0.35V vs. Ag/AgCl in 0.1 M KOH solution for 12,500 s at 1,000 rpm, and the results are shown in Figure 8 (Soren et al., 2016). As expected, CeO_2_/g-C_3_N_4_ exhibited better stability as compared to both Pt/C as well as bare CeO_2_. Moreover, after 12,500 s, relative current value for bare CeO_2_ and Pt/C decreased by 53 and 40% respectively, while in case of CeO_2_/g-C_3_N_4_ composite a 24% decrease in the relative current was observed.

**Figure 9:**
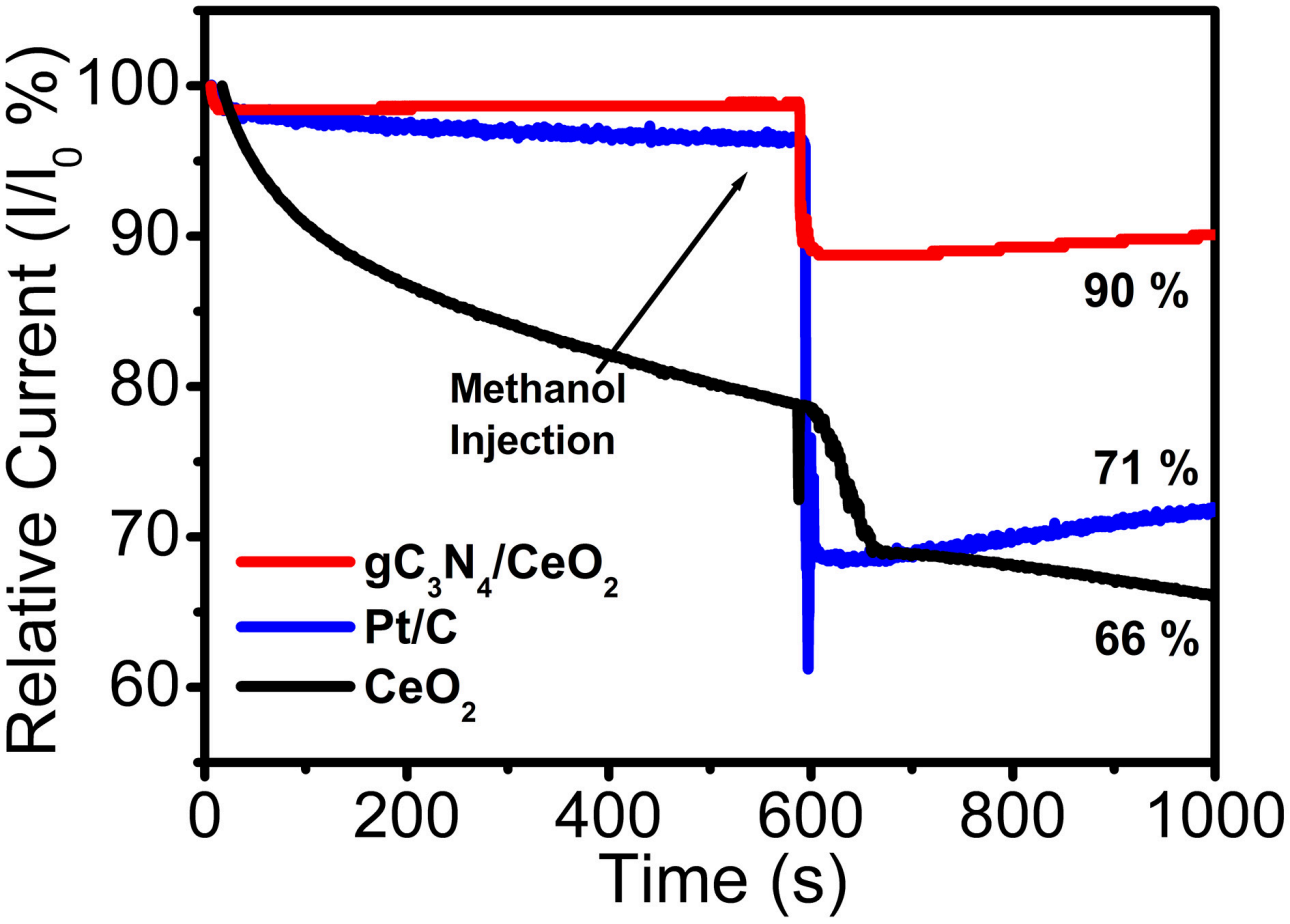
Methanol tolerance test of CeO_2_, CeO_2_/g-C_3_N_4_, and commercial Pt/C.

Methanol poisoning of the cathode impacts the ORR process. As a result, it is very much essential to address another important factor of ORR catalysis—i.e., methanol tolerant capability (Bag et al., 2014). For methanol tolerance, test chronoamperometric measurements were performed at −0.35V vs. Ag/AgCl at 1,000 rpm in O_2_ saturated 0.1 M KOH solution to investigate the methanol crossover effect of CeO_2_, CeO_2_/g-C_3_N_4_ composite as well as of commercial Pt/C. A total of 25 mL of (10 wt%) 3 M methanol was injected at 600 s. It was observed that there was a 34% and 29% decrease in relative current for CeO_2_, Pt/C, respectively, whereas only a 10% decrease in relative current was observed for the CeO_2_/g-C_3_N_4_ composite system (Figure 9). This result demonstrates the better methanol tolerance ability of CeO_2_/g-C_3_N_4_ as compared to Pt/C.

### Impedance Measurement

**Figure 10:**
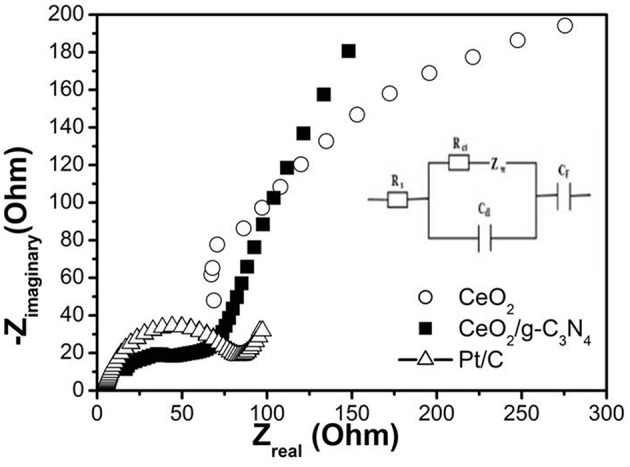
Nyquist plot of as prepared electrocatalyst at −0.21 V vs. Ag/AgCl measured in 0.1 M KOH solution. Inserted: A simplified equivalent circuit.

Electrochemical impedance spectroscopy (EIS) is another potent technique to describe the electrocatalyst kinetics and interface properties in ORR (Perini et al., 2015). EIS analysis through Nyquist plots is generally used to examine interfacial charge transport behavior of the electrode-electrolyte interface. The Nyquist plots demonstrate variation of impedance with frequency reflected as imaginary component vs. real component of impedance. Figure 10 shows the Nyquist plots over the frequency range 100–1 MHz for the CeO_2_/g-C_3_N_4_ modified electrode in 0.1 M KOH solution with an AC amplitude of 0.1 V at an initial potential of −0.210 V. The Nyquist plot is represented at high frequency by a semicircular arc and at low frequency the plot is represented by a straight line. In this plot, the charge transfer resistance at the electrode-electrolyte interface is represented by the diameter of the semicircular arc whereas the diffusion nature of the electrolyte at the electrode surface is represented by a straight line (Parwaiz et al., 2017). The impedance data was fitted to an equivalent circuit (inset of Figure 10). The equivalent circuit consists of charge transfer resistance (R_ct_), solution resistance (R_s_), pseudocapacitance (C_F_), Warburg impedance (Z_w_), and double layer capacitance (C_dl_) (Tan and Ren, 2016). The semicircle intercepting the real axis is a combination of both charge transfer resistance R_ct_ (Guan et al., 2018) and ionic resistance of electrolyte R_s_ (Maheswari and Muralidharan, 2016). R_s_ consists of bulk electrolyte solution resistance and electron transfer resistance whereas R_ct_ can be ascribed to change transfer resistance at the electrode-electrolyte boundary (Maheswari and Muralidharan, 2015).

The calculated R_s_ for the CeO_2_/g-C_3_N_4_ composite is 36.5 Ω. The value of R_s_ of the electrodes can be attributed to the dissimilar conductivities and morphologies of the materials in their construction. A faster electron-transfer rate can be designated by a smaller R_ct_ (Wu Q. et al., 2017). Based on the observations, the diameter of the semicircle and the calculated R_ct_ of the CeO_2_/g-C_3_N_4_ is 150 Ω whereas the R_ct_ for CeO_2_ and Pt/C was found to be 534.4 and 77.3 Ω, respectively. The lower R_ct_ value of CeO_2_/g-C_3_N_4_ can be assigned to active electron transfer kinetics which in turn favor ORR catalytic activity.

## Conclusion

In accordance with the study report, CeO_2_/g-C_3_N_4_ was successfully synthesized by a facile microwave mediated polyol route. The synergic effect of CeO_2_ after interacting g-C_3_N_4_ leads to the enhancement of ORR activity of the CeO_2_/g-C_3_N_4_ modified system as compared to bare CeO_2_. The XPS and ORR kinetics study results reveal that CeO_2_/g-C_3_N_4_ with high levels of pyridinic nitrogen performs better ORR catalytic activity, implying a vital role of pyridinic nitrogen as promoter of ORR. The composites have shown excellent ORR stability and methanol tolerance behavior than commercial Pt/C. The low cost and facile synthesis procedure predict the future utility of the CeO_2_/g-C_3_N_4_ composite in energy conversion system.

## Data Availability

The datasets generated for this study are available on request to the corresponding author.

## Author Contributions

The project is designed by PP. Scientific contributions in term of knowledge and discussion given by KV. SS and IH did the experimental work. DA and AD did the XPS analysis.

### Conflict of Interest Statement

The authors declare that the research was conducted in the absence of any commercial or financial relationships that could be construed as a potential conflict of interest.
